# Causal relationship between nonalcoholic fatty liver disease and different sleep traits: a bidirectional Mendelian randomized study

**DOI:** 10.3389/fendo.2023.1159258

**Published:** 2023-06-02

**Authors:** Zijin Sun, Jing Ji, Ling Zuo, Yifan Hu, Kai Wang, Tian Xu, Qingguo Wang, Fafeng Cheng

**Affiliations:** Synopsis of Golden Chamber Department, Chinese Medicine College, Beijing University of Chinese Medicine, Beijing, China

**Keywords:** NAFLD (nonalcoholic fatty liver disease), insulin, sleep, metabolism, inflammation, Mendelian randomization (MR) analysis

## Abstract

**Background and aims:**

Non-alcoholic fatty liver disease(NAFLD) is common worldwide and has previously been reported to be associated with sleep traits. However, it is not clear whether NAFLD changes sleep traits or whether the changes in sleep traits lead to the onset of NAFLD. The purpose of this study was to investigate the causal relationship between NAFLD and changes in sleep traits using Mendelian randomization.

**Methods:**

We proposed a bidirectional Mendelian randomization (MR) analysis and performed validation analyses to dissect the association between NAFLD and sleep traits. Genetic instruments were used as proxies for NAFLD and sleep. Data of genome-wide association study(GWAS) were obtained from the center for neurogenomics and cognitive research database, Open GWAS database and GWAS catalog. Three MR methods were performed, including inverse variance weighted method(IVW), MR-Egger, weighted median.

**Results:**

In total,7 traits associated with sleep and 4 traits associated with NAFLD are used in this study. A total of six results showed significant differences. Insomnia was associated with NAFLD (OR(95% CI)= 2.25(1.18,4.27), P = 0.01), Alanine transaminase levels (OR(95% CI)= 2.79(1.70, 4.56), P =4.71×10-5) and percent liver fat(OR(95% CI)= 1.31(1.03,1.69), P = 0.03). Snoring was associated with percent liver fat (1.15(1.05,1.26), P =2×10-3), alanine transaminase levels (OR(95% CI)= 1.27(1.08,1.50), P =0.04).And dozing was associated with percent liver fat(1.14(1.02,1.26), P =0.02).For the remaining 50 outcomes, no significant or definitive association was yielded in MR analysis.

**Conclusion:**

Genetic evidence suggests putative causal relationships between NAFLD and a set of sleep traits, indicating that sleep traits deserves high priority in clinical practice. Not only the confirmed sleep apnea syndrome, but also the sleep duration and sleep state (such as insomnia) deserve clinical attention. Our study proves that the causal relationship between sleep characteristics and NAFLD is the cause of the change of sleep characteristics, while the onset of non-NAFLD is the cause of the change of sleep characteristics, and the causal relationship is one-way.

## 1 Introduction

Nonalcoholic fatty liver disease (NAFLD), one of the most common chronic liver disease in the world, affects 25% of the population worldwide and its prevalence is expected to increase further in the near future. NAFLD and its associated consequences have become one of the major public health problems due to changes in lifestyle and dietary structure. NAFLD can be accompanied by many subsequent extensive liver injuries, ranging from simple hepatic steatosis to steatohepatitis, advanced liver fibrosis, cirrhosis and hepatocellular carcinoma (1). Although most patients with NAFLD do not exhibit any symptoms, they may progress to end-stage liver disease or hepatocellular carcinoma, eventually requiring a liver transplant. Epidemiological studies have demonstrated a strong association between the onset of NAFLD and an increased risk of metabolic disorders (2–5),such as obesity, metabolic syndrome and insulin resistance. It is estimated that about 20% of people in China have NAFLD, while in the United States the rate is as high as 35% (6, 7). Therefore, it is crucial to identify possible factors that may influence the development of NAFLD and thus improve lifestyles to reduce the incidence of NAFLD.

Sleep is crucial for general health, and the amount of good sleep is beneficial to health (8). However, in modern society, sleep deprivation is common. The National Sleep Foundation reported in 2013 that one-third of U.S. employees get less than six hours of sleep per day (9), and it is getting worse over time. There is evidence that poor sleep is linked to negative health outcomes (10), such as obesity (11), type 2 diabetes (12), and cardiometabolic disorders (13), and it can increase the risk of NAFLDs’ onset and progression (14, 15). In the previous systematic review and meta-analysis, sleep disruption was reported to alter feeding behavior and timing of food intake, and to alter insulin sensitivity in adipose tissue in human and mouse models (15–17). Because of the strong association between sleep and NAFLD, it is necessary to conduct research on it. However, previous reports on the relationship between the two are often contradictory and inconsistent (14, 18–20), so further investigations and inquiries are needed to clarify the relationship between the two. That is, does sleep changes lead to the development of NAFLD? Or does NAFLD lead to changes in sleep traits?

With the rapid development of genome-wide association studies (GWAS), Mendelian randomization (MR) analysis with phenotype-associated single nucleotide polymorphism(SNP) s as instrumental variables (IVs) is increasingly used in clinical settings to explore the causal relationships between various factors and diseases. Information about genetic variation is used to identify potential effects and to reveal causal relationships between the two. Although randomized controlled trial (RCT) have a high level of evidence, they are often difficult to implement due to their high cost, time consuming nature and sometimes ethical issues involved. In this case, MR provides a useful method, especially when observational studies yield associations with a tendency to bias due to confounding or reverse causality. As an extension of the MR approach, two-sample MR allows aggregated data from GWAS to be used in MR studies, rather than just individual-level data, and without regard to cost. Overall, under the specific assumptions of IVs, a sound MR design can provide more reliable evidence than observational studies (21, 22) that can be used to guide clinical practice.

Based on publicly available GWAS data from a large population, we used a two-sample bidirectional MR analysis to illustrate the effect of sleep on the development of NAFLD, thus further clarifying the causal relationship between sleep and the development of NAFLD. In order to understand the risk factors for NAFLD and provide new insights for the prevention of NAFLD.

## 2 Methods

### 2.1 Research design

In this study, we see insomnia, daytime dozing, morningness, ease of getting up, sleep duration, daytime napping and snoring as sleep-related traits. Since the main traits of NAFLD are steatosis of the liver and impairment of liver function, we used the diagnosis of NAFLD, alanine transaminase levels, aspartate aminotransferase and percent liver fat as a description of NAFLD. In this study, we used a two-way Mendelian randomization approach to assess the causal relationship between sleep and NAFLD.

First, we screened instrumental variables for MR analysis using sleep-related traits as “exposures” and NAFLD and related liver indicators as “outcomes”, and assessed heterogeneity using Cochran Q analysis. Heterogeneity was assessed using Cochran Q analysis, and finally sensitivity analysis was performed to verify the reliability of the causal results. Then, we performed a reverse MR examination, using the diagnosis of NAFLD and related liver indicators as the “exposure” and sleep-related traits as the “outcome”.

### 2.2 Data sources

The GWAS data for insomnia obtained from the Open GWAS database, a dataset published by Neale LABS in 2017 that included a sample of 336,082 participants from European populations. In addition to this, we also used six sleep parameters from the center for neurogenomics and cognitive research database (https://cncr.nl/) as our sleep traits, including daytime dozing (n=386,548),morningness(n=345,552),ease of getting up(n=385,949),sleep duration(n=384,317), daytime napping (n=386,577) and snoring (n=359,916) (23). At the same time, data related to NAFLD were obtained from the Open GWAS database with the GWAS catalog, which included Alanine transaminase levels (n=9,731) (24), Aspartate aminotransferase (n=9,463) (24), Percent liver fat (n=32,858) (25)and NAFLD diagnosis in the electronic medical record (n=377,998) (26). All the data are listed in **Table 1**.

**Table 1:** Details of the GWASs included in the Mendelian randomization.

Phenotype	Participants	Web source
Insomnia	336,965	https://gwas.mrcieu.ac.uk/datasets/ukb-a-13/
Napping	386,577	https://ctg.cncr.nl/documents/p1651/Napping_sumstats_Jansenetal.txt.gz
Dozing	386,548	https://ctg.cncr.nl/documents/p1651/Dozing_sumstats_Jansenetal.txt.gz
Morningness	345,552	https://ctg.cncr.nl/documents/p1651/Morningness_sumstats_Jansenetal.txt.gz
Sleep duration	384,317	https://ctg.cncr.nl/documents/p1651/Sleepdur_sumstats_Jansenetal.txt.gz
Ease of getting up	385,949	https://ctg.cncr.nl/documents/p1651/Gettingup_sumstats_Jansenetal.txt.gz
Snoring	359,916	https://ctg.cncr.nl/documents/p1651/Snoring_sumstats_Jansenetal.txt.gz
Alanine transaminase levels	9,731	https://gwas.mrcieu.ac.uk/datasets/ebi-a-GCST004940/
Percent liver fat	32,858	https://gwas.mrcieu.ac.uk/datasets/ebi-a-GCST90016673/
Aspartate aminotransferase	134,154	https://gwas.mrcieu.ac.uk/datasets/ebi-a-GCST005064/
NAFLD	778,614	https://www.ebi.ac.uk/gwas/publications/34841290

GWA, genome-wide association study, NAFLD, non-alcoholic fatty liver disease.

### 2.3 Selection of instrumental variables

For the selection of IVs, a more relaxed threshold was used in order to include more SNPs, and SNPs that were significant for the whole genome (<5 × 10-7) were used as a selection to become IVs, which has been used in many other MR studies (27). The parameter R^2^ threshold was set to 0.001 and kb was set to 10,000, and the LD_CLUMPING function was merged to exclude the interference of chain imbalance. Missing SNPs were removed from the result database. finally, valid SNPs significantly associated with exposure were obtained as IVs. Weak instrumental variable bias is likely to arise if the correlation between IVs and exposure factors is weak. To avoid weak instrumental variable bias, F values were calculated in this study. The F value is the ratio of the variance explained by the Mendelian randomized first-stage model to the variance of the residuals, and is usually considered to be free of weak instrumental variable bias when F is greater than or equal to 10 (28), using $R^{2}=\left(2EAF\left(1-EAF\right)\right)/\left(2EAF\left(1-MAF\right)+2EAF\left(1-EAF\right)SE^{2}\right)$ (EAF=effective allele frequency, SE=standard error, N=sample size) to calculate the F value. Finally, the data were extracted from the outcome data and collated and merged in order to align the same effect alleles with the exposure and the resulting effect values. Finally, we performed a harmonization process to remove echo SNPs with non-uniform orientation so that their effect alleles remain uniform.

### 2.4 Statistical analysis

As assumed, three assumptions must be satisfied when using the MR method (1): the selected IVs must be strongly associated with the exposure (2); the selected IVs should not be associated with potential confounders (3); the selected IVs could only influence the outcomes through the exposure, but not other pathways We used three different MR analysis methods (random effect inverse variance weighted method (IVW), MR Egger and weighted median method) to assess the causal effects between the two.

In this study, IVW was used as the primary outcome, while the MR-Egger and weighted median method were used to improve IVW estimation, as they can provide more reliable estimates in a wider range of scenarios, albeit less efficient (wider CI). MR-Egger allows all genetic variants to be pleiotropic, but requires pleiotropy to be independent of variant exposure associations (22). The weighted median method allows the use of invalid IVs under the assumption that at least half of the IVs used in the MR analysis are valid. In the IVW analysis, the slope of the weighted regression of SNP outcome effects on SNP exposure effects (where the intercept constraint is zero) represents the outcome estimate. For sensitivity analyses, we then analyzed them using Cochran’s Q test, MR-Egger regression intercept test, and leave-one-out analysis, respectively, with p<0.05 considered statistically significant (22, 29, 30). All analyses we performed using TwoSampleMR (version 0.4.25) with the MR PRESSO (version 1.0) package in R language (version 3.6.1).

## 3 Results

We investigated the correlation of different sleep traits with NAFLD, with the IVW method providing the main results, while the results of the MR Egger and weighted median methods were also presented by us. Processes with P value < 0.05 for the IVW method and F value > 10 were considered as significant associations. We present the statistically significant results in **Table 2**.

**Table 2:** Mendelian randomized analysis of positive results presented.

Exposure	Outcome	Method	P value	OR(95% CI)	Cochran’s Q test P value	MR-Egger intercept derived P value	SNPs
Insomnia	NAFLD	Inverse variance weighted	0.01	2.25(1.18,4.27)	0.04	0.47	49/58
		Weighted median	3×10-3	3.79(1.57,9.13)			
		MR Egger	0.16	4.85(0.56,41.83)			
Insomnia	Alanine transaminase levels	Inverse variance weighted	4.71×10-5	2.79(1.70, 4.56)	0.98	0.87	3/4
		Weighted median	1.60×10-3	2.79(1.47,5.16)			
		MR Egger	1.00	1.00(7.70×10-5,12977.55)			
Insomnia	Percent liver fat	Inverse variance weighted	0.03	1.31(1.03,1.69)	0.01	0.65	55/58
		Weighted median	0.37	1.16(0.85,1.60)			
		MR Egger	0.31	1.61(0.64,4.04)			
Snoring	Percent liver fat	Inverse variance weighted	2×10-3	1.15(1.05,1.26)	0.03	0.40	47/57
		Weighted median	2×10-4	1.23(1.10,1.37)			
		MR Egger	0.26	0.93(0.56,1.55)			
Snoring	Alanine transaminase levels	Inverse variance weighted	0.04	1.27(1.08,1.50)	0.51	0.36	4/7
		Weighted median	0.10	1.20(0.96,1.48)			
		MR Egger	1.00	1.08(0.65,1.53)			
Dozing	Percent liver fat	Inverse variance weighted	0.02	1.14(1.02,1.26)	0.79	0.84	4/4
		Weighted median	0.02	1.15(1.02,1.31)			
		MR Egger	0.53	1.20(0.75,1.93)			

NAFLD, non-alcoholic fatty liver disease, OR, odds ratio, SNP, single nucleotide polymorphism, MR, mendelian randomized.

**Figure 1:**
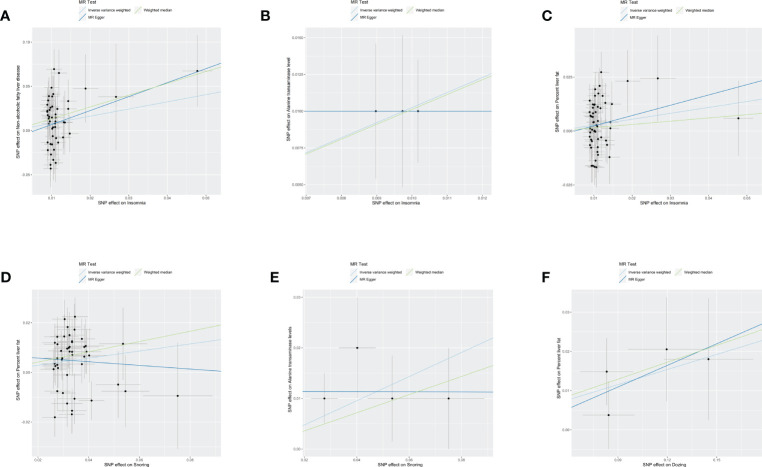
Scatter plots of six resules. The black dot denotes the genetic instrumental variable included in the Mendelian randomization analysis. The grey error bar denotes the 95% confidence interval of the coefficient for each genetic instrumental variable. NAFLD, non-alcoholic fatty liver disease, SNP, single nucleotide polymorphism, MR, mendelian randomized. **(A)** The influence of insomnia on the onset of NAFLD. **(B)** The influence of insomnia on alanine transaminase. **(C)** The influence of insomnia on percent liver fat. **(D)** The influence of Snoring on percent liver fat. **(E)** The influence of Snoring on alanine transaminase. **(F)** The influence of Dozing on percent liver fat.

As shown in the **Table 2**, the existence of multiple sleep traits was statistically significant to the Mendelian randomization analysis that represented the phenotype of NAFLD, especially the trail of insomnia. The scatter plots of six outcomes that showed significant associations are shown in **Figure 1**.

In addition, statistical difference verification of the weighted median method could be obtained for most of the above results (P<0.05).However, we could not obtain any statistically significant results when representing data related to NAFLD as an exposure factor and data related to sleep traits as an outcome factor for reverse causality validation (P<0.05). Although some of the results exhibit heterogeneity when tested using Cochran’s Q test (p>0.05), since we used random effects IVW as the main outcome, the heterogeneity is acceptable and it does not invalidate the MR estimates in the current study (31). And all the results were tested for multiple validity, and the results proved that all the results with significance did not have multiple validity, so we can assume that there is no horizontal multiple validity in this study.

**Figure 2:**
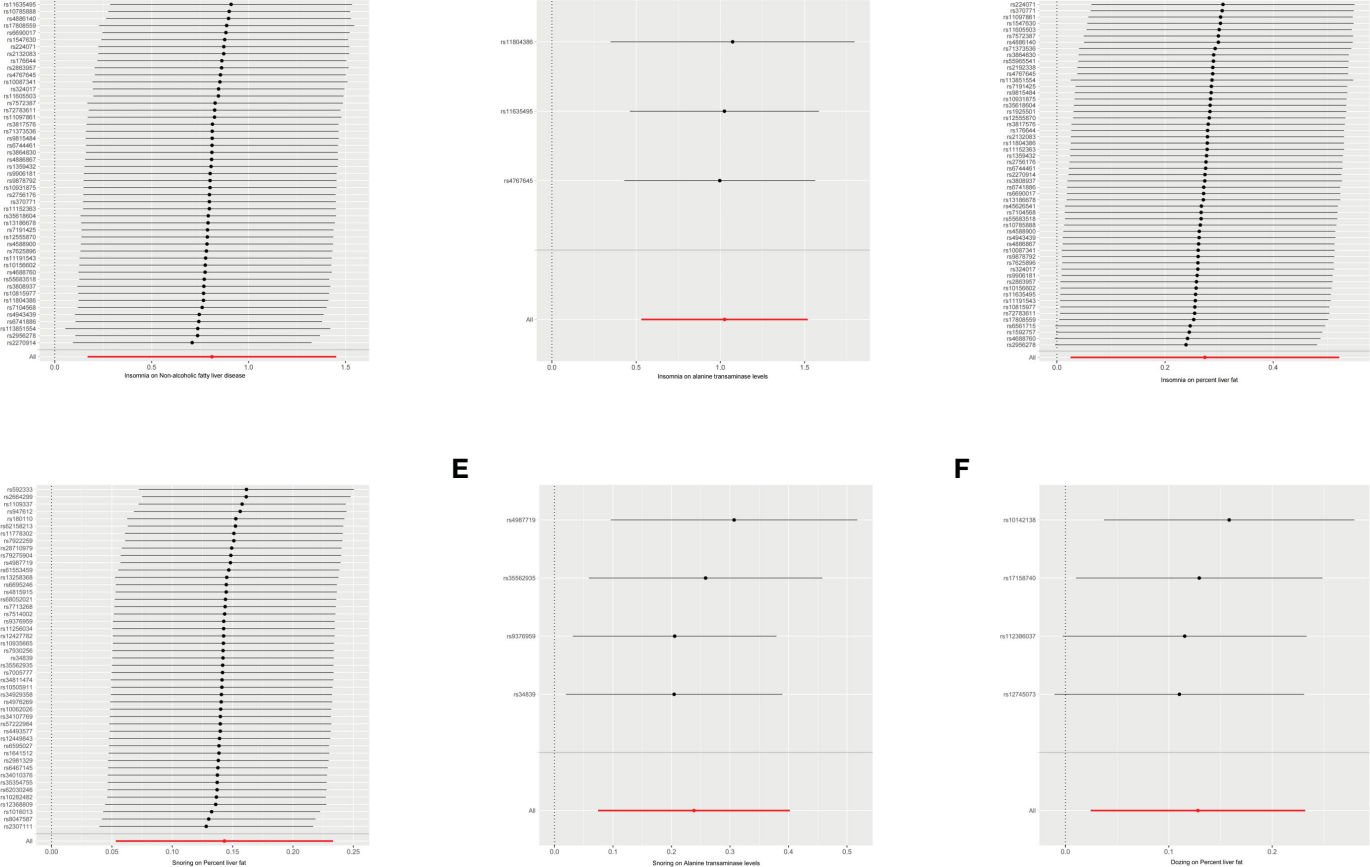
Forest maps of six resules. The vertical axis represents the number assigned to each SNP, while the horizontal axis represents the confidence interval. The red line segment corresponds to the confidence interval category of the entire sample, while the gray line segment corresponds to the confidence interval category of each SNP sample. NAFLD, non-alcoholic fatty liver disease, OR, odds ratio, SNP, single nucleotide polymorphism, MR, mendelian randomized. **(A)** The influence of insomnia on the onset of NAFLD. **(B)** The influence of insomnia on alanine transaminase. **(C)** The influence of insomnia on percent liver fat. **(D)** The influence of Snoring on percent liver fat. **(E)** The influence of Snoring on alanine transaminase. **(F)** The influence of Dozing on percent liver fat.

Next, we perform a sensitivity analysis using the leave-one-out method, which is shown in the forest plot. As seen in the plot, basically all lines are on one side of the y-axis, and even beyond the axis they are not too far away, so it is clear that the results we are exploring are more robust. Forest maps of six results are shown with the results, as shown in **Figure 2**.

## 4 Discussion

Many previous reports have used MR studies to determine the causal relationship between sleep-related problems and various diseases and indicators, such as cardiovascular disease, breast cancer, depression and glycated hemoglobin (32–35). However, the available evidence on sleep and NAFLD is mainly from cross-sectional studies involving reverse causality with contradictory and conflicting findings and opinions (20, 36–41). Therefore, exploring a clear relationship between the two will pave the way for early identification of potential patients, which is essential to achieve earlier monitoring and early diagnosis for effective prevention and treatment.

In the MR analysis of this paper, we systematically evaluated the causal relationship between sleep traits and the onset of NAFLD, and demonstrated that different sleep traits can be the cause of the onset and exacerbation of NAFLD, and NAFLD does not change sleep traits, and the causal relationship between them is unidirectional. In addition to this, all previous observational clinical studies are difficult to avoid confounding risk factors due to the limitations of their own experimental methods, whereas in the present study, MR is less susceptible to measurement error, confounding, and reverse causality than traditional observational studies because the genotypes are robust and formed prior to the exposure factors (42), and with the MR method, we can confidently reveal causality apart from biasing factors.

The pathogenesis of NAFLD is often described as a double whammy model. The first strike is characterized by increased intracellular triglyceride accumulation in hepatocytes due to adipose tissue lipolysis caused by obesity and insulin resistance. The second strike is characterized by the production of lipotoxic metabolites, oxidative stress, lipid peroxidation, mitochondrial dysfunction and the progression of liver inflammation and steatosis due to some genetic polymorphisms (43, 44).

In previous studies, most researchers have targeted the relationship between obstructive sleep apnea(OSA), which is characterized by snoring, and NAFLD. It has been extensively demonstrated experimentally that chronic intermittent hypoxia in OSA may be related to the pathogenesis and severity of NAFLD. In the hypoxic environment of OSA, adipose tissue lipolysis, oxidative stress, inflammation and liver fibrosis are increased (45). Bhatt et al. reported (46) that obese patients with OSA had a significantly increased prevalence of metabolic syndrome and significantly higher levels of interleukin-6, macrophage migration inhibitory factor, high-sensitivity C-reactive protein, and tumor necrosis factor alpha compared to other groups. All these inflammatory biomarkers seem to have an important pathophysiological role in the development of early metabolic and cardiovascular dysfunction. And it has been experimentally demonstrated that animals exposed to intermittent hypoxia develop liver fibrosis with inflammatory liver injury and, when they are exposed to intermittent hypoxia and coupled with another liver injury, they can exhibit significant hepatocyte inflammation and necrosis (47–49).

Therefore, it seems biologically plausible that OSA may exacerbate liver damage in NAFLD, leading to a phenotypic shift toward nonalcoholic steatohepatitis and liver fibrosis. However, the damage caused by OSA in NAFLD is due to hypoxia rather than the effects of sleep itself, which does not fully explain the mechanism of the damage caused by poor sleep habits in the body. Moreover, improving hypoxia alone does not provide a comprehensive treatment for NAFLD.

Although hepatic steatosis is a reversible disease, Continuous Positive Airway Pressure(CPAP) does not seem to improve the morphological changes of the liver in patients with OSA (50, 51). A meta-analysis also concluded that CPAP did not have any effect on liver fibrosis and that the available evidence from the studies was not of high quality to fully demonstrate that CPCA improves liver fibrosis (52).

Therefore, we should focus on the impact that sleep itself has on NAFLD.

In order to clearly understand the mechanisms of sleep, it is necessary to recognize that sleep is part of a 24-hour cycle that is largely regulated by the inevitable cycles of light and darkness. The light and dark cycles due to the rotation of the Earth are the main effect on living things. The periodic alternation of light and dark is the basis of the biological clock, which is ubiquitous in human physiology and behavior. The alignment of sleep and wakefulness with circadian cycles is essential for establishing a healthy sleep cycle. Dysregulation of these environmental changes (e.g., in night shift work) often leads to sleep disorders and pre-sleep symptoms such as insomnia. Fluctuations in cognitive ability and energy over a 24-hour period, as well as the urge to sleep after 14-16 hours of wakefulness, are familiar daily experiences. However, how the biological clock is intertwined with almost every aspect of physiological function, from cells to organs, is not as evident. Well-known physiological cycles include the sleep-wake cycle, the 24-hour temperature cycle, and the secretion of cortisol.

The biological clock plays an important role in metabolic rhythms, such as glucose and lipid homeostasis (53). It was demonstrated that mice with obesity and metabolic syndrome developed after knockout of genes related to biological clock in mice (54–57). Rhythmic changes in insulin sensitivity are altered in part by an autonomous rhythm generated by inputs from the hypothalamus to the liver, with insulin rhythm production regulated by the peripheral beta-cell clock. An experimental study found decreased carbohydrate tolerance in men who went to bed from 1:00 to 5:00 am for six consecutive nights (58). In addition to this, circadian rhythm disturbances would trigger an inter-organ imbalance of sympathetic-parasympathetic branches, and sympathetic overactivity is a recognized risk factor for obesity and insulin resistance, which may further increase the risk of NAFLD (59, 60).

The relevant SNPs obtained from our study also proved our conjecture to some extent. The SNPs we obtained corresponded to a variety of genes, including FTO, RFX3 and KSR2, which are closely related to metabolism and insulin secretion.

For example, FTO expression is increased in NAFLD, and it promotes hepatic steatosis by targeting PPARα (61). Its upregulation can reduce insulin secretion *via* the inflammatory NF-kB pathway and disrupts lipid utilization in skeletal muscles by suppressing the PPARβ/δand AMPK pathways, leading to the occurrence of diabetic hyperlipidemia (62).A recently research found that RFX3 is a novel functional genes that regulate the development of adipose mesenchymal stem cells into islet β-cells (63). KSR2 plays an important role in energy metabolism. In clinical studies, it has been found that patients who lose the function of KSR2 have severe insulin resistance (64, 65). KSR2 interacting with AMPK play significant roles in high insulin level and impaired glucose tolerance which can explain this phenomenon (66).

Insomnia means less sleep, which causes the body to produce more interleukin 6 and tumor necrosis factor, and also activates STAT family proteins (67, 68). In addition, late sleep due to insomnia often leads to certain changes in diet and lifestyle, including more snacking and irregular meals, as well as longer sitting activities, including the use of electronic devices and watching television (69). These behaviors can lead to heat buildup, resulting in NAFLD, which should also be taken seriously.

### 4.1 Strength and limitations

To our knowledge, this is the first bidirectional MR study that provides new insight into the causal associations between sleep traits and NAFLD. Our study has some strengths and limitations. The main advantage of this work is the use of bidirectional MR to explore the causal relationship between sleep traits and NAFLD, which can avoid reverse causality and potential false associations due to confounding factors. Second, all instrumental variables we used were from the publicly available GWAS with a large amount of data, avoiding bias due to anomalies in a few individuals, and none of our instrumental variables were affected by weak instrumental bias (F > 10). Also, we have tested our sensitivities using multiple methods to ensure that the IVs satisfy the core hypotheses and to obtain more robust results.

One of our limitations, however, is that the sleep traits used in this study, particularly the trait of insomnia, do not indicate whether they are self-reported or the result of a clinical diagnosis, and there is some discrepancy between the two (34). Second, we were unable to stratify NAFLD according to its severity and other factors. Third, the data we obtained were mainly from European populations, which may reduce the generalizability of our findings.

### 4.2 Future research

We are thrilled to have found further evidence on the impact of different sleep traits on NAFLD, indicating that enhancing sleep is essential for the prevention and development of NAFLD. In the future, we may explore the specific mechanisms of sleep’s effects on NAFLD, including metabolic and inflammatory pathways, to offer novel insights for the prevention and treatment of NAFLD.

## 5 Conclusion

In summary, our findings suggest that sleep characteristics are associated with an elevated risk of NAFLD. Our study has significant implications for comprehending the causal relationship between sleep characteristics and NAFLD. Further research into their mechanisms and interactions is warranted.

## Data availability statement

The original contributions presented in the study are included in the article/supplementary material. Further inquiries can be directed to the corresponding author.

## Author contributions

ZS, JJ and LZ conceived the study design. KW,YH and TX performed the statistical analysis. ZS wrote the manuscript and performed the data visualization. QW and FC supervised the study. All authors provided critical revisions of the draft and approved the submitted draft.
